# Glucose measurement in cerebrospinal fluid compared to peripheral blood in dogs with central nervous system disease

**DOI:** 10.3389/fvets.2025.1706896

**Published:** 2025-12-17

**Authors:** Megan Wolfe, Lisa Schlein, Jamie Henry, Stephanie McGrath

**Affiliations:** 1 Department of Clinical Sciences, College of Veterinary Medicine and Biomedical Sciences, Colorado State University, Fort Collins, CO, United States; 2 Department of Microbiology, Immunology, and Pathology, College of Veterinary Medicine and Biomedical Sciences, Colorado State University, Fort Collins, CO, United States

**Keywords:** cerebrospinal fluid, spinal tap, meningoencephalitis of unknown origin, blood brain barrier, CSF glucose, inflammatory brain disease

## Abstract

**Introduction:**

Glucose in cerebrospinal fluid (CSF) is an easily measurable parameter that may correlate with inflammatory central nervous system (CNS) disease. The aim of this study was to evaluate CSF glucose concentration and CSF: peripheral blood glucose ratio (CBGR) in healthy dogs of various ages and dogs with inflammatory versus non-inflammatory CNS disease.

**Methods:**

This was a single institution retrospective study. Dogs who had peripheral blood collection within 24 h of CSF tap were included. The CSF glucose measurement and CBGR were calculated for healthy dogs and for dogs with various categories of CNS disease.

**Results:**

Forty-one healthy dogs and 359 dogs with CNS disease were included. Mean (95% CI) CSF glucose in healthy dogs was 78.4 mg/dL (72.0–84.7) and mean CBGR was 0.80 (0.74–0.86). Among healthy dogs, there was a significant positive correlation between age and CBGR (*ρ* = 0.41) (*p* = 0.007). Non-Inflammatory Structural CNS disease had a significantly higher CSF glucose concentration than healthy dogs [87.8 mg/dL (84.2–91.4), *p* = 0.03]. There was no significant difference in mean CBGR compared to healthy dogs for Non-Inflammatory Structural CNS disease [0.81 (0.78–0.84), *p* = 0.98] or Idiopathic Epilepsy [0.75 (0.71–0.79), *p* = 0.28]. Mean CBGR for dogs with Inflammatory CNS disease [0.70 (0.65–0.74)] was significantly lower than that of healthy dogs (*p* = 0.02). When dogs were divided into more specific disease categories, there was no significant difference in CBGR compared to healthy dogs for Autoimmune [0.70 (0.66–0.75), *p* = 0.06], Infectious [0.67 (0.56–0.77), *p* = 0.15], or any other category.

**Discussion:**

Healthy dogs have a CBGR of approximately 0.80, but older age may affect this value. Altered CBGR may help distinguish inflammatory from non-inflammatory structural CNS disease.

## 1 Introduction

Cerebrospinal fluid (CSF) analysis is a key part of the standard veterinary neurological workup. CSF total nucleated cell count (TNCC), protein concentration, and cytological analysis are routinely used for interpretation and provide information about potential underlying autoimmune disease, infection, or neoplasia in the central nervous system (CNS). Research evaluating the utility of glucose in the CSF for more specific diagnosis of various neurological diseases is ongoing. In humans and companion animals, the concentration of glucose within CSF depends on the peripheral blood glucose concentration, the rate of facilitated transport into the CNS, and the overall CNS metabolic rate (1). In healthy human patients, the normal CSF glucose: peripheral blood glucose ratio (CBGR) is approximately 0.60 (2–5). Alterations to this normal ratio may support a diagnosis of CNS pathology such as infectious meningitis (4–8). Other physiologic factors such as patient age may also impact the CBGR (5).

Studies investigating the normal CSF glucose concentration or the CBGR in domestic dogs are extremely limited (1, 9). To the authors’ knowledge, only one recent veterinary study investigated potential changes in CSF glucose level in diseased states. Dogs with steroid-responsive meningitis arteritis (SRMA) and bacterial meningitis previously were reported to have a lower CBGR compared to dogs with idiopathic epilepsy and intervertebral disc disease (IVDD), but there was no significant difference in CBGR between SRMA and bacterial meningitis (9). This suggests that an increase in CSF total nucleated cell count may be responsible for changes in CBGR rather than bacterial catabolism of glucose. However, CBGR for healthy dogs has not been reported in recent literature, and it is unknown if CBGR differs significantly between inflammatory and non-inflammatory structural brain disease compared to healthy dogs. If a relationship between CSF glucose level and CNS inflammation exists, this would be an easily accessible diagnostic test in neurologically affected dogs, given that CSF collection is routinely performed. Additionally, CSF glucose evaluation would be useful when a brain MRI, in the absence of histopathology, yields inconclusive results in dogs with suspected brain neoplasia versus granulomas (10).

The objective of this study was to determine the normal CBGR in healthy dogs of various ages and dogs with inflammatory vs. non-inflammatory CNS diseases. We hypothesized that CSF glucose concentration would correlate with the peripheral blood glucose concentration in a linear fashion. We also hypothesized that increasing CSF TNCC would cause a decrease in the CBGR, similar to what has been suggested by a prior study in dogs (9). A secondary aim was to evaluate whether the CBGR is affected by peripheral blood glucose analytical method.

## 2 Materials and methods

### 2.1 Medical records review

This was a single institution retrospective observational study. The medical records system for Colorado State University Veterinary Teaching Hospital was reviewed for all dogs who had CSF collected from the cerebellomedullary cistern from 1/1/2014 through 1/31/2023. All clients of the Veterinary Teaching Hospital sign a treatment consent form authorizing the use of medical records for research and publication; therefore, additional consent was not required for the present study.

Dogs were included if a peripheral blood glucose measurement (serum biochemistry or venous blood gas) was obtained within 24 h of CSF tap. Serum biochemistry samples were analyzed using a Cobas c501 chemistry analyzer (Roche Diagnostics, Rotkreuz, Switzerland). Venous blood gas samples were analyzed using an ABL800 or ABL90 venous blood gas analyzer (Radiometer, Brea, CA, USA). All machines at Colorado State University received routine maintenance and quality control testing. Point of care glucometer was not used for any included glucose measurement.

For included dogs, the following data were collected from the medical record: age, sex, breed, comorbidities, clinical diagnosis, CSF TNCC (#/uL), CSF protein concentration (mg/dL), CSF red blood cell count (RBC, #/uL), CSF glucose concentration (mg/dL), CSF cytologic interpretation, peripheral blood glucose (mg/dL), and method of peripheral glucose measurement (serum biochemistry vs. venous blood gas analyzer). Due to the retrospective nature of the study, specific individual anesthetic and MRI protocols were not standardized or recorded. All dogs had general anesthesia using a patient-specific anesthetic protocol approved by a board-certified anesthesiologist. The MRI scans were all performed with a 1.5 Tesla (T) (Siemens MAGNETOM Altea, Malvern, PA, USA) or 3.0 T (Siemens Skyra, Malvern, PA, USA) magnet. All brain MRI scans included a minimum of T2-weighted (T2-W) sagittal, T2-W transverse, Fluid-Attenuated Inversion Recovery (FLAIR) transverse, and T1-W transverse pre- and post-contrast (Gadolinium, 0.1 mmol/kg IV) sequences. All vertebral column MRI scans included a minimum of T2-W sagittal, T2-W transverse, and T1-W transverse sequences, with post-contrast (Gadolinium, 0.1 mmol/kg IV) added as per primary clinician discretion.

Dogs were excluded if medical records were incomplete, if the CSF collection site was the lumbar subarachnoid space or the collection site was not clearly documented, or if the dog had diabetes mellitus. If the cause of the patient’s disease could not be clearly determined based on review of the medical record, or the patient had more than one clinically significant CNS disease (i.e., brain tumor and a history of idiopathic epilepsy), these patients were excluded. Exposure to glucocorticoid therapy was not recorded, as this has previously been shown not to correlate with CSF glucose in dogs, and effects on glucose transport into the brain are expected to be minimal in patients with normoglycemia (9, 11). If the same patient had multiple CSF taps performed over time, only the first for which complete data was available was included; repeat CSF samples were excluded.

Based on review of the medical record, each case was assigned to a subgroup of neurological disease. These included: Idiopathic Epilepsy, Inflammatory CNS Disease, Non-inflammatory Structural CNS Disease, and Other. Dogs in the Inflammatory CNS disease category were further categorized into Infectious and Autoimmune. Dogs were assigned to the “Infectious” diagnosis category if the medical record indicated that bacterial or fungal organisms were seen on CSF analysis, if infectious disease serologic testing and/or cultures (blood, urine, or CSF) were positive, or if the primary clinician strongly suspected an infectious etiology based on the patient history, MRI findings, and response to treatment. Cases were assigned to the “Autoimmune” diagnosis category if the medical record indicated that signalment, MRI and CSF findings, and negative infectious disease testing (when submitted) were most consistent with an autoimmune etiology. Dogs in the Non-Inflammatory Structural CNS disease category were further categorized into CNS Neoplasia, Intervertebral Disc Disease, and Vascular event. Histopathology was reviewed when available. In the absence of histopathology, a presumptive diagnosis of Meningoencephalitis of Unknown Origin (MUO), SRMA, Infectious disease, CNS Neoplasia, Intervertebral Disc Disease, and Vascular event was assigned based on the diagnosis indicated by the primary clinician in the medical record.

Control data was obtained from healthy client-owned dogs that were voluntarily enrolled as controls in another study within the same time period, with approval to use banked CSF for additional research purposes (CSU IACUC #4106). The control dogs had no recorded history of seizures or other abnormal neurological behavior. All control dogs had a normal neurological examination performed by a board-certified veterinary neurologist, or a veterinary neurology resident or intern under the supervision of a board-certified veterinary neurologist. All healthy control dogs had routine hematology and serum biochemistry panel, followed by brain MRI and cisternal CSF tap under general anesthesia within 24 h of peripheral blood sampling. Brain MRI and CSF analysis were normal in all control dogs. No control dogs were diagnosed with diabetes mellitus. No control dogs were noted to be receiving systemic glucocorticoids; other medications and specific anesthetic protocols were not recorded.

All CSF samples were collected into a glass tube without additive and processed within 30 min of collection. The CSF TNCC and RBC were counted by trained laboratory personnel using a manual manual Bright-Line hemocytometer (Hausser Scientific, Horsham, PA, USA). The CSF protein and glucose measurement were determined using a Cobas c501 chemistry analyzer (Roche Diagnostics, Rotkreuz, Switzerland) by the automated urine/CSF protein method with Benzethonium chloride (12). A cytospin preparation was made by combining 1 drop of 22% bovine albumin with 1 drop (50uL) of CSF, and centrifuging at 50G force for 4 min using a Shandon Cytospin 4 centrifuge (Thermo Fisher, Waltham, MA, USA). The cytospin slide was then stained with Wright-Giemsa stain for microscopic examination and nucleated cell differential count performed by a veterinary clinical pathologist.

### 2.2 Statistical analysis

Commercially available software was used for statistical analysis (JMP® Pro 18.0.2; SAS Institute Inc., Cary, North Carolina). Model assumptions of normality were evaluated by visual inspection of diagnostic plots. Normally distributed continuous data (patient age, CSF glucose, peripheral blood glucose, CBGR) are reported as mean (95% CI). Non-normally distributed continuous data (CSF TNCC, CSF protein, CSF RBC count) are reported as median [interquartile range (IQR)].

To evaluate the impact of peripheral blood sampling method (Serum biochemistry vs. venous blood gas analyzer), a pooled t-test was used to determine if there was a significant difference in mean peripheral blood glucose or CBGR of samples measured with these two analytical methods for each disease category. One way analysis of variance (ANOVA) was used to compare mean CSF glucose measurement and mean CBGR amongst disease categories. Because the “Other” category included a wide variety of diverse conditions, data was presented but these dogs were not included for comparison with other groups. Dunnett’s test was used as a post-hoc analysis to identify which groups were significantly different from the control group (healthy dogs). A *p* value of <0.05 was considered significant. Spearman’s rank test was used to assess the correlation between CSF TNCC and the CSF glucose, CSF TNCC and CBGR, and patient age and CBGR.

## 3 Results

### 3.1 Case selection and demographic information

Initially 673 dogs were identified by medical records review. Of these, 433 dogs met the inclusion criteria. An additional 74 dogs were excluded due to unclear diagnosis (*n* = 47), multiple CNS diseases (*n* = 9), repeat CSF samples from the same dog (*n* = 8), incomplete medical records (*n* = 7), or diabetes mellitus (*n* = 3); leaving 359 dogs with CNS disease which were included for analysis. Forty-one healthy control dogs meeting the inclusion criteria were identified.

The mean age at time of CSF tap was 79 months (range 2–204 months) for dogs with CNS disease, and 79 months (range 19–190) for healthy dogs. Distribution of dogs into the various disease categories is shown in Table 1. “Autoimmune” diagnoses included MUO (*n* = 44), SRMA (*n* = 16), and Corticosteroid Responsive Tremor Syndrome (*n* = 4). “Infectious” diagnoses included bacterial (*n* = 10), fungal (*n* = 1), and viral (*n* = 1) etiologies. “Neoplasia” diagnoses included dogs with presumptive primary (*n* = 51) or secondary (*n* = 7) neoplastic lesions. Neurological conditions of dogs in the “Other” diagnoses group are listed in Table 2.

**Table 1:** Demographic information and blood analytical method for included dogs.

Category of disease		Number of patients	Age (months)	Sex	Peripheral blood analysis method
Healthy dogs (*n* = 41)		41	79 (19–190)	MC = 20 (48.8%) MI = 1 (2.4%) FS = 19 (46.3%) FI = 1 (2.4%)	SBC: 41 VBG: 0
All dogs with CNS disease (*n* = 359)		359	79 (2–204)	MC = 137 (38.2%) MI = 35 (9.7%) FS = 159 (44.3%) FI = 28 (7.8%)	SBC: 185 VBG: 189
Inflammatory CNS disease (*n* = 77)	Autoimmune	64	57 (2–155)	MC = 16 (25%) MI = 8 (12.5%) FS = 30 (46.9%) FI = 10 (15.6%)	SBC: 33 VBG: 36
	Infectious	13	35 (3–98)	MC = 4 (30.8%) MI = 3 (23.1%) FS = 3 (23.1%) FI = 3 (23.1%)	SBC: 6 VBG: 8
Non-inflammatory CNS disease (*n* = 127)	Neoplasia	58	109 (8–186)	MC = 22 (37.9%) MI = 2 (3.4%) FS = 30 (51.7%) FI = 4 (6.9%)	SBC: 25 VBG: 36
	IVDD	48	101 (24–197)	MC = 24 (50%) MI = 4 (8.3%) FS = 19 (39.6%) FI = 1 (2.1%)	SBC: 31 VBG: 16
	Vascular	21	82 (29–144)	MC = 13 (61.9%) MI = 1 (4.8%) FS = 7 (33.3%) FI = 0 (0%)	SBC: 7 VBG: 14
Idiopathic epilepsy (*n* = 94)		94	59 (5–192)	MC = 39 (41.5%) MI = 9 (9.6%) FS = 42 (44.7%) FI = 4 (4.3%)	SBC: 49 VBG: 48
Other (*n* = 61)		61	95 (2–205)	MC = 19 (31.2%) MI = 8 (13.1%) FS = 28 (45.9%) FI = 6 (9.9%)	SBC: 34 VBG: 31

Age is reported as mean (range). CNS, Central nervous system; IVDD, Intervertebral Disc Disease; MC, Male castrated; MI, Male intact; FS, Female spayed; FI, Female intact; SBC, Serum biochemistry; VBG, Venous blood gas.

**Table 2:** Dogs with neurological disease categorized as “Other.”

Diagnosis	Number of patients	CBGR
Idiopathic vestibular disease	19	0.74 (0.48–0.87)
Congenital malformation	10	0.78 (0.63–1.03)
Canine cognitive dysfunction	6	0.74 (0.61–0.87)
Cervical spondylomyelopathy	5	0.85 (0.67–0.92)
Degenerative lumbosacral stenosis	5	0.93 (0.77–1.31)
Idiopathic cranial polyneuropathy	4	0.69 (0.68–0.70)
Trauma	4	0.74 (0.55–0.92)
Toxin	2	0.67 (0.59–0.76)
Synovial cyst	2	0.77 (0.60–0.93)
Metabolic disease	2	0.75 (0.60–0.89)
Polyradiculoneuritis	1	0.88
Storage disorder	1	0.73

CBGR is reported as mean (range). CBGR, CSF:Blood glucose ratio.

### 3.2 Peripheral glucose measurement with serum biochemistry vs. venous blood gas

Of the 359 dogs with CNS disease, 170 had serum biochemistry submitted within 24 h of CSF collection, 174 had venous blood gas analysis, and 15 had both serum biochemistry and venous blood gas submitted (Table 1). There were 207 dogs who had peripheral blood glucose measured on the same day as CSF collection, and 152 dogs who had peripheral blood glucose measured the day prior to CSF collection. Further analysis of the exact timing of peripheral blood collection relative to the timing of CSF collection was not performed.

**Figure 1:**
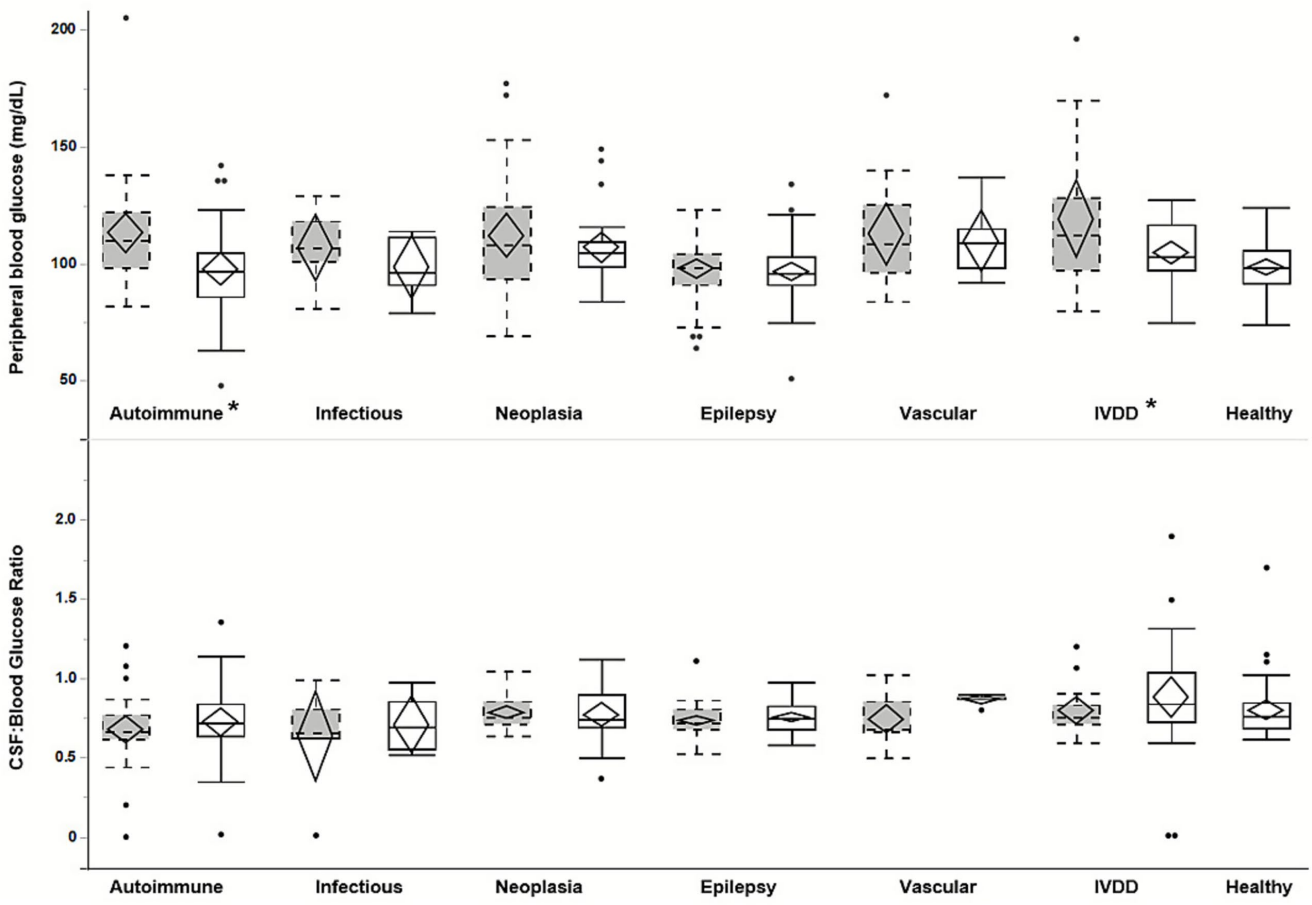
Box plot and confidence diamond for the peripheral blood glucose measurement and CSF: Blood Glucose Ratio for each disease category, divided by analytical method. Grey boxes with dashed outlines represent venous blood gas measurements, and solid boxes represent serum biochemistry analyzer measurements. An asterisk (*) represents a significant difference between peripheral blood analytical methods for a given category (*p* < 0.05). IVDD, Intervertebral Disc Disease.

Table 3 summarizes the mean peripheral blood glucose and CBGR for each specific disease category and for healthy dogs. Within the “Autoimmune” diagnoses category, the one-way ANOVA test found a significant difference in mean peripheral blood glucose measurement based on peripheral blood analytical method (*p* = 0.005), however there was no significant difference in the mean CBGR based on analytical method (*p* = 0.42). Within the “IVDD” category, there was a significant difference in mean peripheral blood glucose measurement (*p* = 0.03), however there was no significant difference in the mean CBGR (*p* = 0.37). For every other disease category, one-way ANOVA determined that the method of peripheral blood analysis did not significantly affect the mean peripheral blood glucose measurement or the mean CBGR (Figure 1). For the 15 dogs with both serum biochemistry and venous blood gas submitted, there was no significant difference in the peripheral blood glucose level (*p* = 0.17) or the CBGR (*p* = 0.16) between the two sampling methods. Therefore, all 359 samples were included for comparison to healthy controls, regardless of method of peripheral blood glucose analysis. For the 15 dogs that had both a serum biochemistry and venous blood gas submitted, the serum biochemistry glucose value was used for analysis.

**Table 3:** Clinicopathologic information for each specific category of disease.

Category	CSF TNCC (#/uL)^*^	CSF Protein (mg/dL)^*^	CSF RBC (#/uL)^*^	CSF glucose (mg/dL)^‡^	Blood glucose (mg/dL)^‡^	CBGR^‡^
Healthy (*n* = 41)	2 (1–3)	19.0 (14.0–26.0)	4 (1–70)	78.4 (72.0–84.7)	98.8 (93.1–104.5)	0.80 (0.74–0.86)
Autoimmune (*n* = 64)	44 (6–299)	41.1 (19.0–82.4)	74 (7–707)	73.3 (68.2–78.3)	105.3 (100.8–109.9)	0.70 (0.66–0.75)
Infectious (*n* = 13)	5 (1–1,114)	23.3 (14.0–96.5)	222 (4–412)	69.0 (57.7–80.3)	103.2 (93.1–113.3)	0.67 (0.56–0.77)
Neoplasia (*n* = 58)	2 (1–8)	27.5 (18–62.4)	15 (1–260)	84.9 (79.6–90.2)	110.0 (105.3–114.8)	0.78 (0.73–0.83)
IVDD (*n* = 48)	2 (1–4)	21.5 (11.2–29.8)	8 (1–154.8)	91.2 (85.4–97.1)	109.7 (104.4–114.9)	0.86 (0.80–0.91)
Vascular (*n* = 21)	2 (1–4)	19.5 (12.3–30.8)	13 (1–40)	88.1 (79.3–97.0)	111.8 (103.9–119.8)	0.78 (0.70–0.87)
Epilepsy (*n* = 94)	1 (0–2)	15.6 (13–19.8)	3 (0–36.5)	72.2 (68.0–76.4)	97.4 (93.7–101.2)	0.75 (0.71–0.79)

Non-normally distributed data (*) are reported as median (IQR). Normally distributed data (‡) are reported as mean (95% CI). CSF, Cerebrospinal fluid; TNCC, Total nucleated cell count; RBC, Red blood cell count; CBGR, CSF:Blood glucose ratio; IVDD, Intervertebral disc disease.

### 3.3 Comparison of CSF glucose and CBGR in healthy dogs and dogs with neurologic diseases

**Figure 2:**
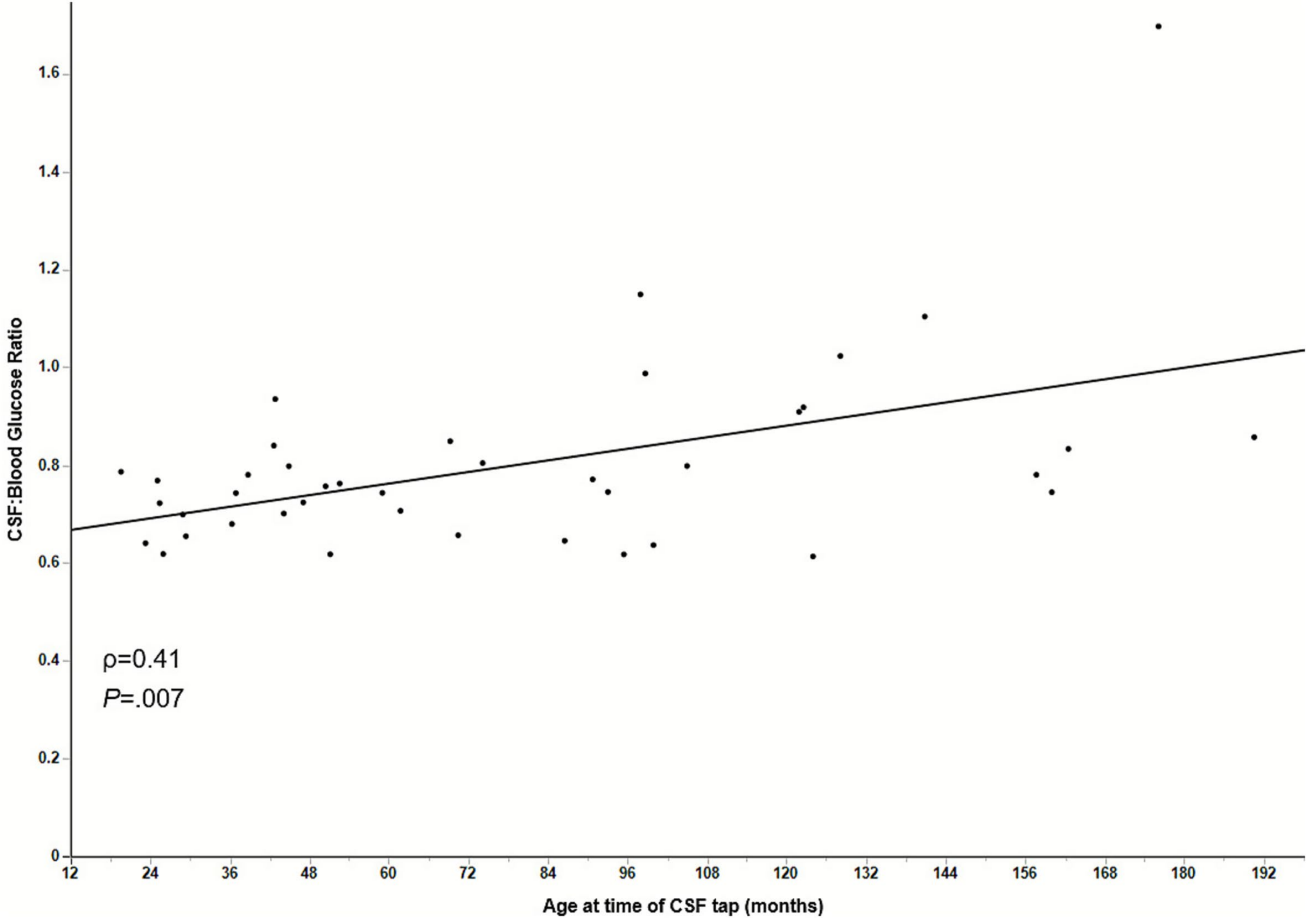
Scatter plot and line of best fit showing a significant positive Spearman correlation between patient age and CBGR in healthy control dogs. CSF, Cerebrospinal fluid.

Healthy control dogs had a mean CSF glucose measurement of 78.4 mg/dL (95% CI, 72.0–84.7). The mean CBGR in healthy dogs was 0.80 (95% CI, 0.74–0.86). Among the 41 healthy dogs, there was a significant positive correlation between age and CBGR (*ρ* = 0.41) (*p* = 0.007), with older healthy dogs having a higher mean CBGR than younger healthy dogs (Figure 2).

**Figure 3:**
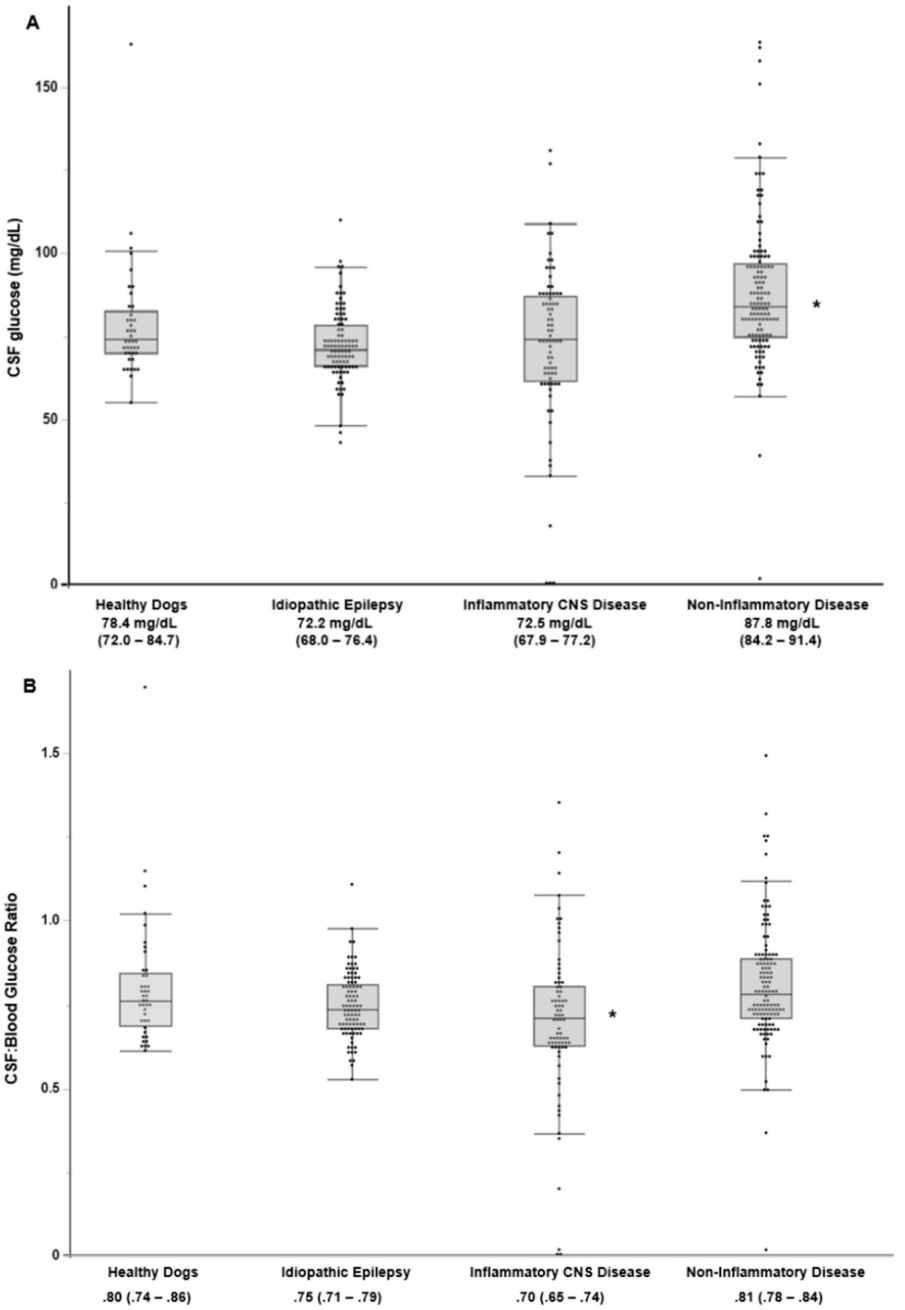
Box plot of the **(A)** CSF glucose and **(B)** CBGR for each broad disease category. For each category, the mean (95% CI) is listed. An asterisk (*) indicates a significant difference from healthy controls using Dunnett’s post-hoc analysis (*p* < 0.05). CNS, Central Nervous System.

On ANOVA with Dunnett’s post-hoc analysis, the only broad category of disease with a significant difference in the mean CSF glucose measurement compared to healthy dogs [78.4 (72.0–84.7)] was Non-Inflammatory Structural CNS disease [87.8 (84.2–91.4), *p* = 0.03]. No significant difference in the mean CSF glucose concentration was found for Idiopathic Epilepsy [72.2 (68.0–76.4)] or Inflammatory CNS Disease [72.5 (67.9–77.2)] when compared to healthy dogs (Figure 3A). The only broad category of disease with a significant difference on post-hoc analysis in the mean CBGR compared to healthy dogs [0.80 (0.74–0.86)] was Inflammatory CNS disease [0.70 (0.65–0.74), *p* = 0.02]. No significant difference in the mean CBGR was found for Idiopathic Epilepsy [0.75 (0.71–0.79), *p* = 0.28] or Non-Inflammatory Structural CNS disease [0.81 (0.78–0.84), *p* = 0.98] when compared to healthy dogs using Dunnett’s post-hoc analysis (Figure 3B).

**Figure 4:**
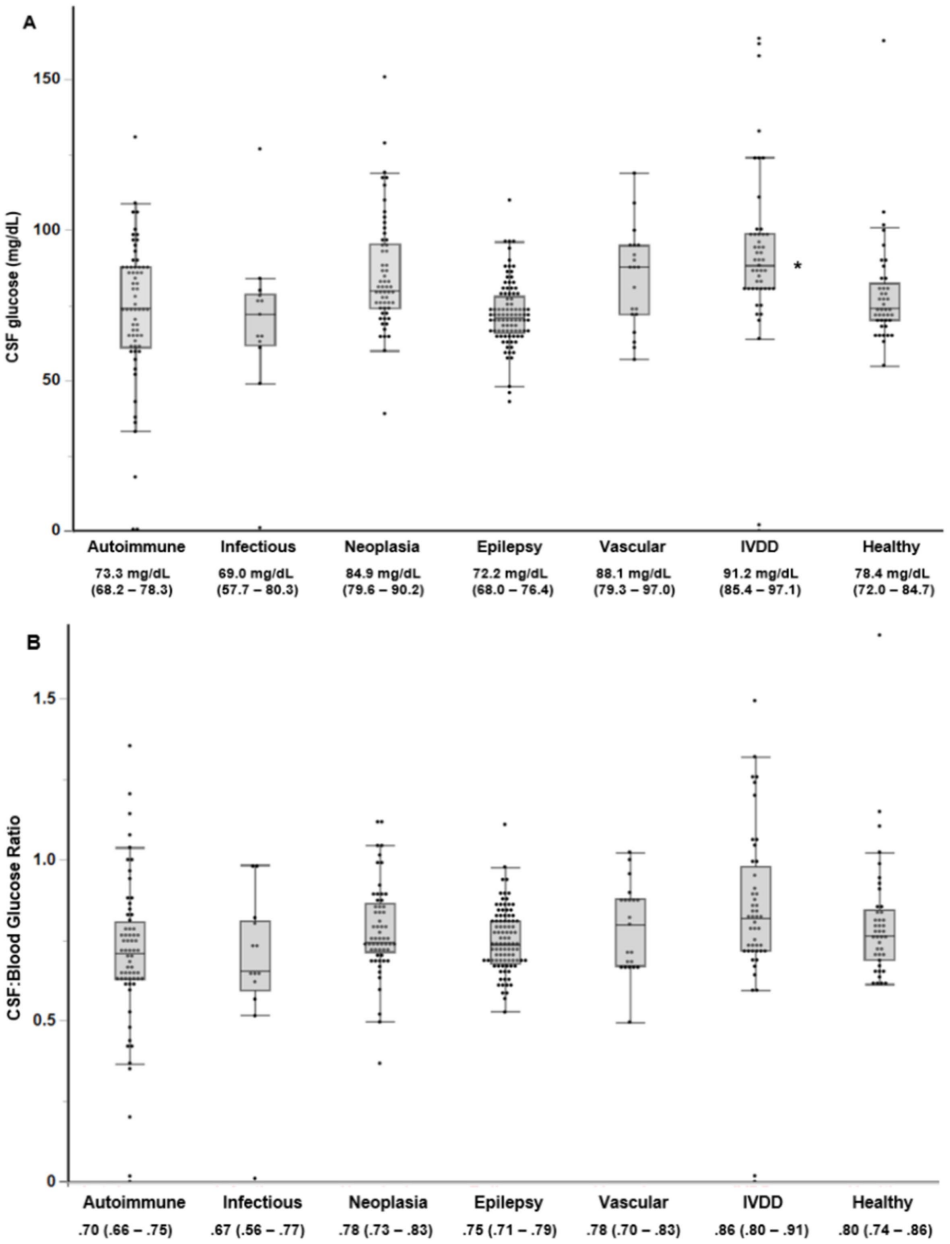
Box plot of the **(A)** CSF glucose and **(B)** CBGR for each specific disease category. For each category, the mean (95% CI) is listed. An asterisk (*) indicates a significant difference from healthy controls using Dunnett’s post-hoc analysis (*p* < 0.05). IVDD, Intervertebral Disc Disease.

When assessing the more specific disease groups, Dunnett’s post-hoc analysis found that the only category with a significantly different mean CSF glucose measurement from Healthy dogs [78.4 (72.0–84.7)] was IVDD [91.2 (85.4–97.1), *p* = 0.02] (Figure 4A). ANOVA found a significant difference in the mean CBGR between the specific disease categories (*p* = 0.001). However, no significant difference in the mean CBGR compared to healthy dogs was found for Autoimmune [0.70 (0.66–0.75), *p* = 0.06], Infectious [0.67 (0.56–0.77), *p* = 0.15], or any other specific disease group on Dunnett’s post-hoc analysis (Figure 4B).

For all 400 dogs (both healthy and diseased, including the “Other” category), there was a weak positive correlation between CSF TNCC and CSF glucose measurement (ρ = 0.02), but this correlation was not significant (*p* = 0.75). There was a significant small negative correlation between CSF TNCC and CBGR (*ρ* = −0.14) (*p* = 0.004). This suggests that the CBGR decreased as CSF TNCC increased, regardless of the underlying diagnosis.

## 4 Discussion

This study supports that normal CBGR in healthy dogs is approximately 0.80. In the present study, decreasing CBGR correlated with the clinical diagnosis of inflammatory CNS disease, and there was a small negative correlation between CBGR and TNCC regardless of underlying disease. Patient age positively correlated with CBGR in healthy controls, with older dogs having a slightly higher CBGR compared to younger dogs. The effect of age on CBGR has also been demonstrated in human medicine (5); reasons for this are unclear and warrant further investigation with larger prospective cohorts.

Studies in human medicine have identified hypoglycorrhachia in bacterial meningitis, CNS disease due to other infectious agents (viral, fungal, protozoal, amoebic), CNS malignancy, hemorrhagic stroke, glucose transporter 1 (GLUT1) receptor deficiency, and non-infectious (aseptic) meningitis (6). Hypoglycorrhachia appears less common in human cases of aseptic meningitis compared to infectious causes, with the exception of neurosarcoidosis (8). The exact pathophysiology behind hypoglycorrhachia is unclear. Proposed mechanisms include dysfunction of the glucose transporter molecules in diseased states, increased glucose uptake by leukocytes during phagocytosis, and excessive glucose metabolism by infectious organisms (4–8, 13). Bacteria such as *E. coli* have been shown to downregulate central nervous system proteins implicated in GLUT1 expression in neonatal meningitis (14).

In this study, infectious disease appeared to lower the CBGR relative to autoimmune disease, but neither of these categories had a mean CBGR that was significantly different from control dogs on post-hoc analysis. Post-hoc analysis only evaluated for a significant difference in mean CBGR for disease categories compared to healthy control dogs; the specific pairwise comparison of mean CBGR for Autoimmune disease versus mean CBGR for infectious disease was not evaluated. A previous study in dogs specifically compared SRMA and bacterial meningoencephalomyelitis, and no significant difference in CBGR was found between these two groups (9). This may indicate that infectious CNS disease has less of an impact on glucose metabolism in dogs than in humans, or that glucose uptake into the CNS is regulated by different mechanisms. Alternatively, this may be a result of the small number of dogs diagnosed with infectious disease resulting in Type II error. This highlights the need for larger-scale, multicenter studies to further investigate the effect of infectious organisms on CSF glucose metabolism.

Many factors can affect glucose metabolism including patient breed, age, and diet (15). Even within the same individual patient there is some inherent variability in peripheral blood glucose measurement (16). This highlights the utility of CBGR instead of the CSF glucose measurement alone as a marker of neurological disease, since it is less influenced by external and metabolic factors. In the present study, the effect of specific anesthetic drugs or systemic medications such as anti-seizure medications on CSF glucose measurement is unknown. Anesthetics including ketamine, propofol, isoflurane, and xylazine may inhibit GLUT1-mediated glucose transport (17, 18). Given that CSF collection in domestic animals necessitates general anesthesia, the effect of anesthetic drugs on CSF glucose measurement is difficult to avoid. Additionally, the use of glucocorticoids was not recorded as a factor in this study. Glucocorticoids increase insulin resistance and reduce glucose uptake in peripheral tissues, leading to hyperglycemia. This partially serves to preserve plasma glucose for optimal neuronal function in times of stress (19). The specific effects of glucocorticoids on GLUT1 receptors in the brain are unclear, and it is unknown if any patients had glucose metabolism affected by glucocorticoids. Patients with peripheral hyperglycemia were excluded from this study, so the effects of systemic glucocorticoids on glucose metabolism in this patient population are expected to be minimal, but this cannot be confirmed.

Timing of sample collection and processing can also affect glucose measurement (20). At our institution, all samples are typically processed within 1 h and therefore the effects of improper handling on glucose measurement are expected to be negligible. Due to the retrospective nature of the study, exact timing of peripheral glucose measurement relative to the CSF tap was not standardized. In all included dogs, peripheral blood glucose was measured within 24 h of CSF tap. This includes samples collected prior, during, and following general anesthesia. The fed state of the patient was not routinely documented in the medical records, so it could not be confirmed if there was a significant difference between fasted and post-prandial samples; however, the clinical significance of fed state on glucose measurement is suspected to be minimal (21). As stated earlier, the decision was made to include samples collected within 24 h of CSF tap so as not to create bias towards dogs who underwent emergency MRI and CSF tap the same day as presentation. It has been shown that venipuncture performed before versus after CSF tap does not significantly affect the peripheral blood glucose measurement or CBGR in children (22). However, CSF glucose may take several hours to equilibrate with the peripheral glucose (2), which may explain why some dogs had a much higher CBGR with CSF glucose even being greater than peripheral blood glucose in some dogs.

For 15 dogs with paired samples available, there was no significant difference in CBGR when analyzing peripheral blood glucose by serum biochemistry or venous blood gas. Furthermore, our results suggest that while analytical method (serum biochemistry panel vs. venous blood gas analysis) affected the peripheral blood glucose measurement for some categories, the method of peripheral blood analysis did not have a significant impact on the CBGR for any category. This suggests that either serum biochemistry or venous blood gas analysis can be used to obtain a peripheral glucose measurement for calculation of the CBGR. However, future prospective studies would ideally standardize both the method and timing of peripheral glucose collection relative to CSF collection. Other factors such as the effect of red blood cell count on glucose measurement were not evaluated. Previous studies have not identified a clear relationship between blood contamination and CSF TNCC (23, 24), but the potential effects of RBC on glucose warrant further investigation.

Another limitation related to the retrospective nature of the study is the chance of misdiagnosis. While dogs were excluded if a single diagnosis was not clear based on review of the medical record, it is possible that some dogs were miscategorized. Previous studies have demonstrated the potential for misdiagnosis of up to 12% of gliomas as infarcts and up to 35% of infarcts as neoplastic lesions (25). Ideally histopathology would be performed in all dogs, but this would have significantly reduced case numbers and likely would have biased the study towards more severe dogs that did not survive. Additionally, it should be noted that the categories “Autoimmune,” “Infectious,” and “Neoplasia” included dogs with multiple different specific etiologies, which may be viewed as a limitation of the study. Finally, it should be noted that the study results should only apply when CSF is collected from the cerebellomedullary cistern, as this was the case for all dogs in the present study. It is unknown if results would vary when CSF is collected from the lumbar subarachnoid space; this would require further prospective research.

In conclusion, the ratio of CBGR is approximately 0.80 in healthy dogs, though this may vary with patient age. A decrease in the CBGR in patients with CNS disease may indicate the presence of CNS inflammation, though this change is nonspecific. Further studies are needed to elucidate if CSF glucose ratio can distinguish between infectious and autoimmune meningoencephalomyelitis.

## Data Availability

The original contributions presented in the study are included in the article/supplementary material, further inquiries can be directed to the corresponding author.
